# Evaluation of the effectiveness and equity of the maternity protection reform in Chile from 2000 to 2015

**DOI:** 10.1371/journal.pone.0221150

**Published:** 2019-09-11

**Authors:** Iris Delgado, Baltica Cabieses, Mauricio Apablaza, Carla Castillo, Ximena Aguilera, Isabel Matute, Manuel Najera, Juan M. Pericàs, Joan Benach

**Affiliations:** 1 Centro de Epidemiología y Políticas de Salud, CEPS, Facultad de Medicina Clínica Alemana Universidad del Desarrollo, Santiago, Chile; 2 Programa de Estudios Sociales en Salud, Instituto de Ciencias e Innovación en Medicina, ICIM, Universidad del Desarrollo, Santiago, Chile; 3 Centro de Políticas Públicas, Facultad de Gobierno, Universidad del Desarrollo, Santiago, Chile; 4 Grupo Desigualdades en Salud (GREDS-EMCONET), Departamento Ciencias Políticas y Sociales, Universitat Pompeu Fabra, Barcelona, España; 5 JHU-UPF Public Policy Center, Universitat Pompeu Fabra, Barcelona, España; 6 Grupo de Investigación Transdisciplinar sobre Transiciones Socioecológicas (GinTRANS2), Universidad Autónoma de Madrid, Madrid, España; Western Oregon University, UNITED STATES

## Abstract

**Introduction:**

According to the International Labor Organization, Maternity Protection (MP) policies try to harmonize child care and women's paid work, without affecting family health and economic security. Chile Law 20.545 (2011) increased benefits for economically active women and reduced requirements for accessing these benefits. The goals of the reform included: 1) to increase MP coverage; and 2) to reduce inequities in access to the benefits.

**Method:**

This study uses two data sources. First, using individual data routinely collected from 2000 to 2015, yearly MP coverage access over time was calculated. Second, using national representative household surveys collected before and after the Law (2009 and 2013), coverage and a set of measures of inequality were estimated. To compare changes over time, we used non-experimental, before-after intervention design for independent samples. For each variable, we estimated comparative proportions at 95% confidence interval before and after the intervention. Additionally, we included multivariate and propensity score analysis.

**Results:**

Between 2000 and 2015, MP coverage grew from 24.4% to 44.8%. Using comparable 2009 and 2013 survey data, we observed the same trend, with 31.6% of estimated MP coverage in 2009, escalating to 39.5% in 2013. We conclude that: 1) after the reform, there was an increase in MP coverage; and, 2) there was no significant reduction of inequities in the distribution of MP benefits.

**Discussion/Conclusion:**

Few scientific evaluations of MP reforms have been conducted worldwide; even fewer including an equity analysis. This study provides an empirically-based evaluation of MP reform from both a population-level and an equity-focused perspective. We conclude that this reform needs to be complemented with other policies to ensure maternity protection in terms of access and equity in a country with deep socioeconomic stratification.

## Introduction

Since 1919, the International Labor Organization (ILO) has promoted Maternity Protection (MP), and, later, MP was declared “a fundamental human right” [1]. ILO established that the MP has two goals: “to preserve the health of the mother and her newborn, and to provide a measure of job and income security (protection from dismissal and discrimination, the right to resume work after leave, and maintenance of wages and incomes during maternity)” [1]. To this end, in 2000, the General Conference of ILO defined five core elements of MP: “1) maternity leave: the woman’s right to a period of rest from work in relation to pregnancy, childbirth, and the postnatal period; 2) cash and medical benefits: the mother’s right to cash benefits during her absence for maternity and health care related to pregnancy, childbirth, and postnatal care; 3) health protection at the workplace for the mother and unborn child during pregnancy, as well as during breastfeeding; 4) employment protection and non-discrimination; 5) breastfeeding arrangements to help workers breastfeed or express milk at the workplace” [2].

Even though experts agree on the importance of MP policies for increasing the social and economic well-being of countries, in 2014, there were still 830 million women workers across the world who had no access to these benefits, 80% of whom were living in low-income countries [1].

Over the last 40 years, many countries have reformed their MP legislation and benefits, especially in connection with the length of paid maternity leave and the introduction or extension of parental leave for fathers [3]. In fact, in Latin America, between 2003 and 2013, five countries (Argentina, Uruguay, Colombia, Brazil, and Chile) implemented policy reforms and modified their legal framework related to MP [4].

In the scientific research literature, there is agreement regarding the impact of maternal leave on mothers' health: longer prenatal and postnatal leave periods associated with better physical and mental health in mothers [5–7], the length of breastfeeding, and children’s health and development [8–11]. Also, there is evidence about the possible impact of MP reforms on broader social indicators, such as employment, income, and women's fertility rates [12–17]. A recent paper by Albagly and Rau explores the causal effects of the Chilean reform in cognitive abilities of children, breastfeeding duration, maternity leave, and employment opportunities after motherhood [18].

On the other hand, several reports have described pending global challenges in this regard, such as the family-work balance, standardizing relevant terminology, and comparing the legal framework and benefits provided in various countries [1, 18–21]. However, there are few evaluations of the fulfillment of the explicit objectives proposed by these policies. To our knowledge, the work of Ekberg analyzing the 1995 Swedish reform (Daddy-Month) is one of the few examples where the objective of the law, which aimed to increase a father’s involvement in child care, was evaluated. They concluded that fathers took the benefit, but did not necessarily take care of their children [22].

### Maternity protection in Chile

In Chile, maternity benefits were introduced in 1917 for working women (Law 3.186), and by 1973, almost all ILO benefits were included in the Chilean legislation. Law 17.928 (1972) established the following mandatory and irrevocable benefits for all working women meeting legal requirements: (1) a six week leave before partum and another 12 weeks after partum with full remuneration covered by the state, up to a maximum of $3,100 USD (present value); (2) paid leave for the mother with a sick child under 1 year of age; (3) a two year job protection from the beginning of pregnancy (the woman cannot be fired); (4) and one hour daily breastfeeding break [23].

In spite of the institutional efforts, the percentage of all pregnant women who benefited from MP was low. Additionally, the distribution of MP benefits was pervasively unequal, with most recipients being women in upper income quintiles [4, 24, 25].

Law 20.545 was promulgated in 2011 to overcome this low coverage, to reduce the unequal MP coverage access, and to stimulate father´s participation in child care. To achieve these goals, the law increased the benefits and reduced the legal requirements in order to extend the MP benefits to the vulnerable population with a focus on those women in informal employment (e.g., workers without a formal contract or with temporary employments). Regarding the benefits, the 2011 reform increased maternal leave from 12 to 24 weeks (30 weeks if women return to their job on a part-time basis) and incorporated a father’s paid leave of up to 5 weeks instead of the mother’s, under the same conditions. Access to MP was simplified in two ways: 1) women no longer require a contract when they get pregnant, and 2) the minimum period of previous contributions to social security was reduced from three months to one month [26, 27].

During the Congressional debate, there was an intense discussion concerning the coverage and inequities of access to MP. This reform was expected to reduce inequities between different socioeconomic groups of working women, with focus on those who earned less and were less educated. No attention was paid to non-working pregnant women in any socioeconomic class, and this group was excluded from the reform´s target population.

The literature shows strong evidence of beneficial impacts that extend maternal leave on the health impact of mother and children [5–11]. However, it is also relevant to analyze the magnitude of access to these benefits, a point little explored by researchers. Knowing about the access to benefits is important, since understanding the access to coverage is helpful for designing strategies in order to implement more effective policies.

Our study complements results from previously cited Albagli and Rau. Our work focuses on the specific objectives of the Law instead of the impacts of the reform, namely, changes in the coverage and inequality. Following the WHO guiding framework for monitoring and evaluation of intervention, we evaluated outcome indicators rather than impact indicators, with a focus on equity [28].

This paper evaluates the effectiveness of Chilean MP in terms of its two main objectives: 1) to increase the coverage of MP; and 2) to reduce inequities of access to MP benefits. Due to data limitations, the third objective of the law is not addressed in this study.

## Methods

We performed a non-experimental, before-after intervention design. The intervention was the implementation of Law 20.545 in 2011, which modified the requisites and benefits associated with maternity protection policies in Chile.

First, using routinely collected individual data from 2000 to 2015, yearly MP coverage access over time was calculated. The main outcome was MP coverage, defined as the number of women who had paid postpartum leave in a certain year, divided by the number of women who gave birth to a live child the same year.

Second, using a nationally representative household surveys collected before and after the Law (2009 and 2013), coverage and a set of measures of inequality were estimated. We studied the distribution of MP coverage across socioeconomic variables of women who gave birth.

To compare changes over time, we used a non-experimental, before-after intervention design for independent samples. For each variable, we compared proportions estimated at 95% confidence interval before and after the intervention. Additionally, we included a multivariate and propensity score analysis using socioeconomic and sociodemographic confounders.

### Information sources

To assess MP coverage, we used data for the number of women with maternal leave after birth between 2000 and 2015 published yearly by the Social Security Superintendency (Superintendencia de Seguridad Social, SUSESO) [29]. SUSESO oversees the administration of financial resources assigned to each beneficiary from the public and private system. The register is based on routinely collected data obtained from individual monthly payments for maternal protection benefits. We also used the number of women who gave a live birth, using official data from the Department of Health Care Statistics and Information (Departamento de Estadísticas e Información en Salud, DEIS), at the Ministry of Health [30]. In Chile, each newborn is registered by the authority, and multiple births (e.g., twins) were adjusted to one women. All data used to calculate the MP coverage are based on administrative information that, in the case of Chile, is compulsory and provides the most accurate and complete source of data in this matter for the country.

To analyze equitable access to MP benefits, we used information from the National Socioeconomic Characterization Survey (CASEN), which provides information on the socioeconomic level of the Chilean population, commonly absent in administrative data sources. CASEN is a periodic survey initiated in 1987, conducted by the Ministry of Social Development, and it is representative of the entire Chilean population. Sampling design is probabilistic, multistage, and stratified by urban/rural areas, with independent samples for each measurement, collecting information from the head of the household on all household members, including children[31]. In our study, CASEN 2009 and 2013 surveys were the points of reference for the before and after comparison of the MP Law that was implemented in 2011. In 2009, 71,460 households were visited, with 246,924 residents of all ages, of which 50.9% were women. Correspondingly, in 2013, 66,725 households were visited, interviewing 218,491 people; 52.3% were women. Anonymized CASEN databases can be retrieved from the Ministry of Social Development website [32–33].

#### Dependent variable

There are two measures of MP coverage because there are two objectives, and each measure corresponds to a different objective: 1) “MP coverage” was calculated with official databases from SUSESO and DEIS; 2) “Estimated MP coverage” was created using CASEN survey data. At the individual level, each woman in the sample was assigned a 0 or 1 following the procedure below:

- With full access to the MP benefits. Value “1” was assigned to women fulfilling three conditions: 1) she had a hospital discharge for vaginal delivery or cesarean section; 2) she was part of the labor force; and, 3) she contributed to social security during the month prior to participation in CASEN survey. This condition is mandatory, whether women are in a formal or informal job.
- Without access to MP benefits. Value “0” was assigned to women who, having had a hospital discharge for delivery, are not part of the labor force or did not contribute to social security during the month prior to participation in CASEN survey (S1 Fig).

#### Independent variables

Several subsets of relevant sociodemographic and socioeconomic subgroups have been defined to explore differences across them. There is a vast literature exploring the existence of health inequities between groups of different socioeconomic levels. Some relevant variables, frequently used as socioeconomic indicators are: educational level, household income, and type of health insurance. Moreover, a key aspect in the analysis of universal coverage of a policy is related to the even and equitable access across subgroups.

The variables selected for these analyses include:

**Intervention variable:**

- Assigns value “0” for women who gave birth in 2009 (before reform) and “1” for those who gave birth in 2013 (after reform). For analytical purposes, we considered this outcome for 2009 and 2013 surveys, separately, at all times.

**Sociodemographics:**

- Age groups: “15–19 years”, “20–29 years”, “30–39 years”, “40 years and older”;
- Area of residence: “urban”, “rural”;
- Marital status: “Yes” (married/cohabiting) — “No” (single/separated/divorced/ widowed);
- Native population: “Yes”, (people who identify themselves or descend from any of the native population groups that the Law recognizes in Chile) — “No”.

**Socioeconomics:**

- Type of Health insurance: “Public” and “Private”
- Education level: “Primary” (< = 8 years), “Secondary” (9–12), “Higher” (> = 13 years);
- Household income quintile: “1 (poorest)”, “2”, “3”, “4”, and “5 (richest)”;
- Multidimensional poverty index (MPI): “Poor”, “No Poor”; The MPI is a national official measure to capture vulnerability comprising information of 12 non-monetary indicators organized in four dimensions of wellbeing: education, access to health, employment and housing. The multidimensional poverty measure (MPI) complements traditional income indicators providing a broader perspective of the individual´s wellbeing. Following the Sen Capability approach and using the Alkire and Foster method, the MPI identifies each household as poor if four or more of the indicators are not satisfied [34, 35].

#### Data analysis

“MP Coverage” was calculated for every year from 2000 to 2015 using census data; consequently, confidence intervals are meaningless. On the other hand, “Estimated MP coverage” was calculated for the years 2009 and 2013 using survey data, thus, confidence intervals are provided for each of those yearly cases.

In order to compare and measure inequalities associated with MP access, a range of methods were applied. To assess the difference between the estimated MP coverage before and after the law, we used a bivariate analysis by sociodemographic and socioeconomic variables. We verified the statistical significance of the difference using a Chi-squared test for independent samples. In addition, we used an inverse probability of treatment weighting, using the propensity score to compare the coverage and controlling for socioeconomic and sociodemographic variables. All confounders were selected a priori based on literature on inequality, and inter-correlations were assessed by Spearman’s correlation.

Inequality levels were estimated using three procedures before and after the intervention: 1) relative range; 2) concentration index and concentration curve (Lorenz curve); and 3) odds ratios.

The relative range provides an intuitive measure of inequality by measuring the relative disparity between the most advantaged and the least advantaged subgroup. The concentration index is a relative measure of inequality that shows how an indicator of interest (coverage by MP) is distributed across the entire population ranked by socioeconomic status, potentially concentrating among the advantaged or disadvantaged. This distribution is usually illustrated using a concentration curve that, in the X-axis, ranks the individuals (women with child) by socioeconomic status (autonomous household income) and their cumulative fraction of MP coverage (Y axis). The concentration index is calculated as twice the area between this concentration curve and the line of equality (45-degree line). A concentration curve lies below the 45-degree line if the indicator of interest is concentrated among the advantages. The concentration index (CI) is a continuous variable that ranges between -1 and +1. A positive value of the CI represents a distribution of the outcome of interest that concentrates among the most advantaged population. These measures provide a more robust graphical and statistically equivalent alternative, respectively, to compare the complete distribution of outcomes and not only to indicate relative gaps between extreme socioeconomic groups [34, 35]. Finally, we estimated odds ratios for each socioeconomic characteristic, using a logistic regression controlled by socioeconomic and sociodemographic variables.

We used a test of difference of proportions for independent samples to assess changes in inequality using the relative range indicators and the concentration index. All analyses were conducted applying standardized weights, based on the complex sampling procedure of CASEN survey [36] with STATA V12 (StataCor, TX, USA). Statistical significance was set at a 95% confidence interval and p<0.05.

## Results

The trend for the number of women who gave birth to a live child birth has been stable from 2000 to 2015 in Chile, with a slight decrease from 243,158 to 242,038 (1.9%) between extreme years of the period. The MP coverage during this period increased from 24.4% to 44.8%.

**Fig 1:**
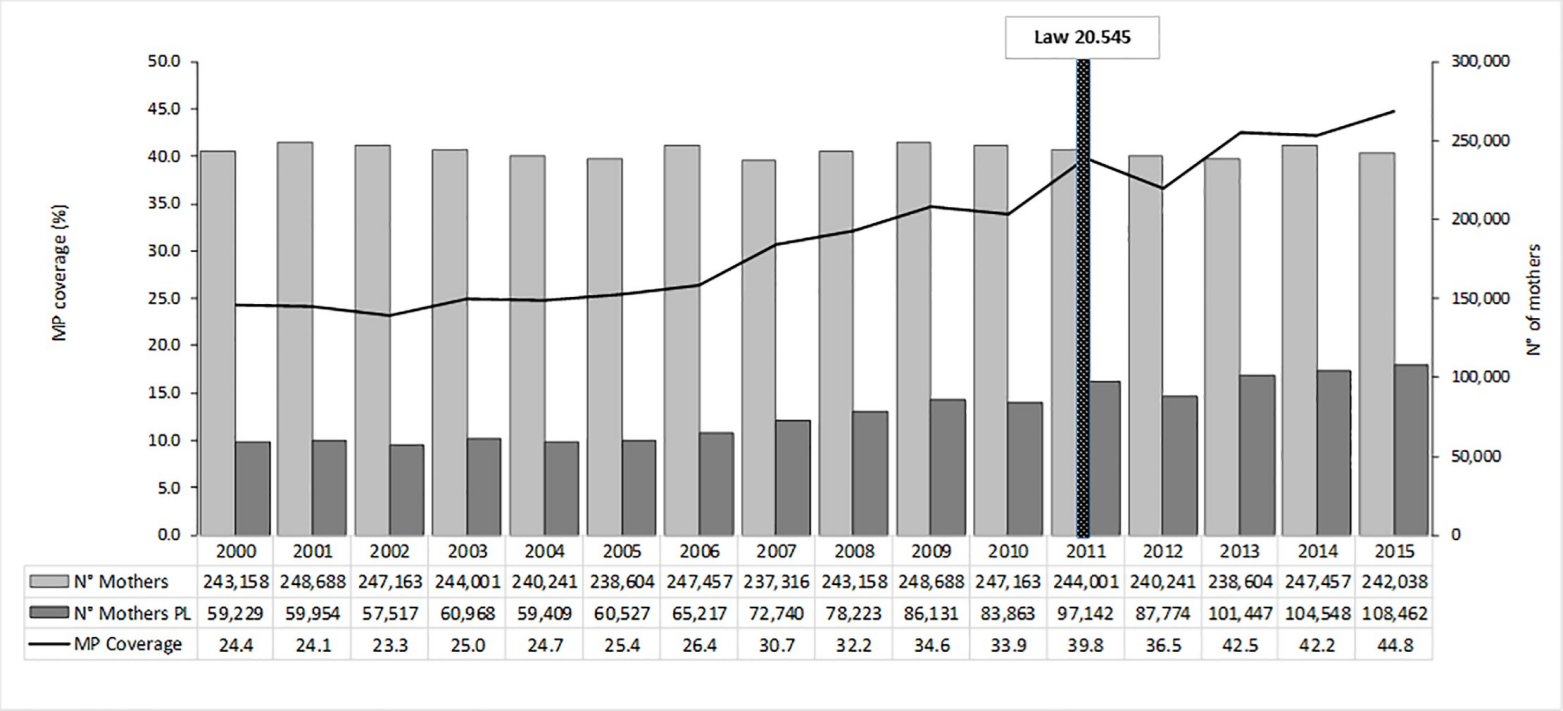
Evolution of maternal protection coverage (2000–2015).

Fig 1 shows the evolution of the number of women who gave birth to a live child (“N° Mothers”); the number of women who took postnatal leaves (“N° Mothers PL”), and the “MP Coverage” across the years 2000 to 2015 in Chile.

On the other hand, the “Estimated MP coverage” improved from 31.1% to 39.5% (p<0.001) between 2009 and 2013. (Table 1). It should be noted that MP Coverage is within the range of the CI for Estimated MP Coverage, representing a fairly similar estimate compared to the MP coverage gold standard.

**Table 1:** MP coverage, estimated MP coverage according to MP coverage.

	Pre-Law (2009)	Post-Law (2013)	P value
MP Coverage	34,60	42,50	
Estimated MP Coverage % (IC95%)	31.1(27.5–34.9)	39.5(36.1–43.0)	0.001(a)

(a) Chi-squared test. Compared Estimated MP Coverage rates before and after the law

### Estimated MP coverage according to sociodemographic variables

In 2009, the mean age for mothers was 27.5 years, increasing to 28.6 years in 2013. Additionally, women who accessed MP were, on average, 4 to 5 years older than those who did not access this benefit. Even though a significant increase in the estimated MP coverage was observed in women aged 30–39 (p<0.01), coverage decreased in younger women (15–19 years old) and in older women (40–49 years old), respectively.

Women from urban areas had a significant advantage in access to MP before and after the reform, showing a significant increase from 32.8% to 41.3%. In the case of rural working women, there was a non-significant increase from 19.5% to 25.7%. By marital status, there was a significant increase for both those mothers in a relationship and the single mothers, from 32.8% to 40.4%, and 27.6% to 37.6% respectively. However, more mothers in a relationship use MP after the law´s enforcement. Another relevant factor was belonging to the native population, and these mothers did not show a relevant improvement. In comparison, access for non-native women jumped from 31.6% to 41.0% of MP coverage, which represented a significant change over time (Table 2).

**Table 2:** Estimated maternity protection coverage according to sociodemographic variables (2009 and 2013).

Variables		Estimated Maternity Protection Coverage		P value (a)
		Before Law (2009)	After Law (2013)	
		% (95% CI)	% (95% CI)	
Age range in years	15–19	4.5 (1.8–11.1)	4.0 (1.6–9.4)	0.83
	20–29	28.7 (24.0–33.9)	33.6 (28.5–56.4)	0.12
	30–39	44.2 (37.3–51.2)	56.4 (50.6–62.0)	**0.01**
	40–49	47.7 (33.1–62.7)	39.2 (28.4–51.1)	0.38
Area of residence	Urban	32.8 (28.8–37.0)	41.3 (37.6–45.1)	**0.00**
	Rural	19.5 (14.2–26.3)	25.7 (19.8–32.6)	0.18
Marital status	Yes	32.8 (28.2–37.8)	40.4 (36.1–44.8)	**0.02**
	No	27.6 (22.7–33.1)	37.7 (32.0–43.8)	**0.01**
Native population	Yes	23.0 (13.2–37.1)	26.7 (19.1–36.1)	0.63
	No	31.6 (27.9–35.5)	41.0 (37.3–44.7)	**0.00**

(a) Chi-squared test. Compared Estimated MP Coverage rates before and after the law

A propensity score was estimated using a logistic regression. The dependent variable, a binary indicator, captures females with maternity protection. We included the same confounders used in the previous model and the estimation only included individuals without missing data (293,112 weighted cases corresponding to 3,912 observations). Our results suggest a significant increase in number of females who used the MP: 3.6% with p<0.001.

### Estimated MP coverage according to socioeconomic variables

Women with higher and middle level of education showed a non-significant improvement in the estimated MP coverage, with a reduction in the coverage among less educated women. This represented an increase in the relative range when comparing low education with high education groups, from 2.8 times pre-reform to 5.5 times more likely to access MP post-reform among well educated women compared to less educated working women.

Estimated MP coverage was higher for women who ascribed to private health insurance than those in the public scheme in both years. However, between 2009 and 2013, there was a significant increase in MP coverage for women who had public health insurance, growing from 26.3% to 33.6%. This rate of increase was not reflected in benefits for those in the private system.

In the case of privately insured working women, data showed rates of 59.9% to 71.0% before and after the Law, respectively. In addition, the relative range according to the type of healthcare provision entitlement was reduced from 2.3 to 2.1 between these groups.

Regarding income, MP coverage increased across all quintiles, but improvement was significant only for the middle-income quintile 3, where it rose from 32.3% to 44.0% before and after the reform. The relative range between the richest quintile and poorest quintile decreased from 8.5 to 6.8. Nevertheless, the MP coverage remained highly unequal between quintiles 5 and 1 after the MP Law, 75.6% and 11.1%, respectively.

Concerning multidimensional poverty, the MP coverage for working women living in multidimensional poverty dropped from 20.5% to 19.0%; whereas, coverage rose significantly among non-poor mothers from 37.6% to 46.7% between the two periods. There was an increase in the relative range according to multidimensional poverty before and after the reform, from 1.8 to 2.5 (Table 3).

**Table 3:** Estimated maternity protection coverage according to socioeconomic variables (2009 and 2013).

Variables		Estimated Maternity Protection Coverage		P value (a)
		Before Law (2009)	After Law (2013)	
		% (95% CI)	% (95% CI)	
Education level	Low	18.8(11.1–29.9)	11.2(7.4–16.6)	0,12
	Mid	25.2(21.3–29.4)	30.1(26.0–34.5)	0,11
	High	52.9(44.7–61.0)	61.2(55.6–66.6)	0,10
Relative range (high/low)		2.8	5.5	
Health care system	Public	26.3(22.8–30.2)	33.6(30.3–37.1)	0,01
	Private	59.9(46.9–71.6)	71.0(61.6–78.9)	0,15
Relative range (Private/Public)		2.3	2.1	
Income quintile	1 (poorest)	8.6(5.7–12.8)	11.1(8.4–14.5)	0,31
	2	24.2(18.6–30.8)	31.3(25.6–37.7)	0,11
	3	32.3(26.0–39.3)	44.0(36.3–52.0)	0,03
	4	50.8(41.4–60.1)	60.1(51.6–68.0)	0,15
	5 (richest)	72.7(58.5–83.5)	75.6(65.3–83.7)	0,71
Relative range (richest quintile/poorest quintile)		8.5	6.8	
Multidimensional poverty	Poor	20.5(15.9–25.9)	19.0(14.3–24.8)	0,69
	Non-poor	37.6(32.8–42.5)	46.7(42.8–50.7)	0,01
Relative range (Non-poor/Poor)		1.8	2.5	

(a) Chi-squared test. Compared Estimated MP Coverage rates before and after the Law

**Fig 2:**
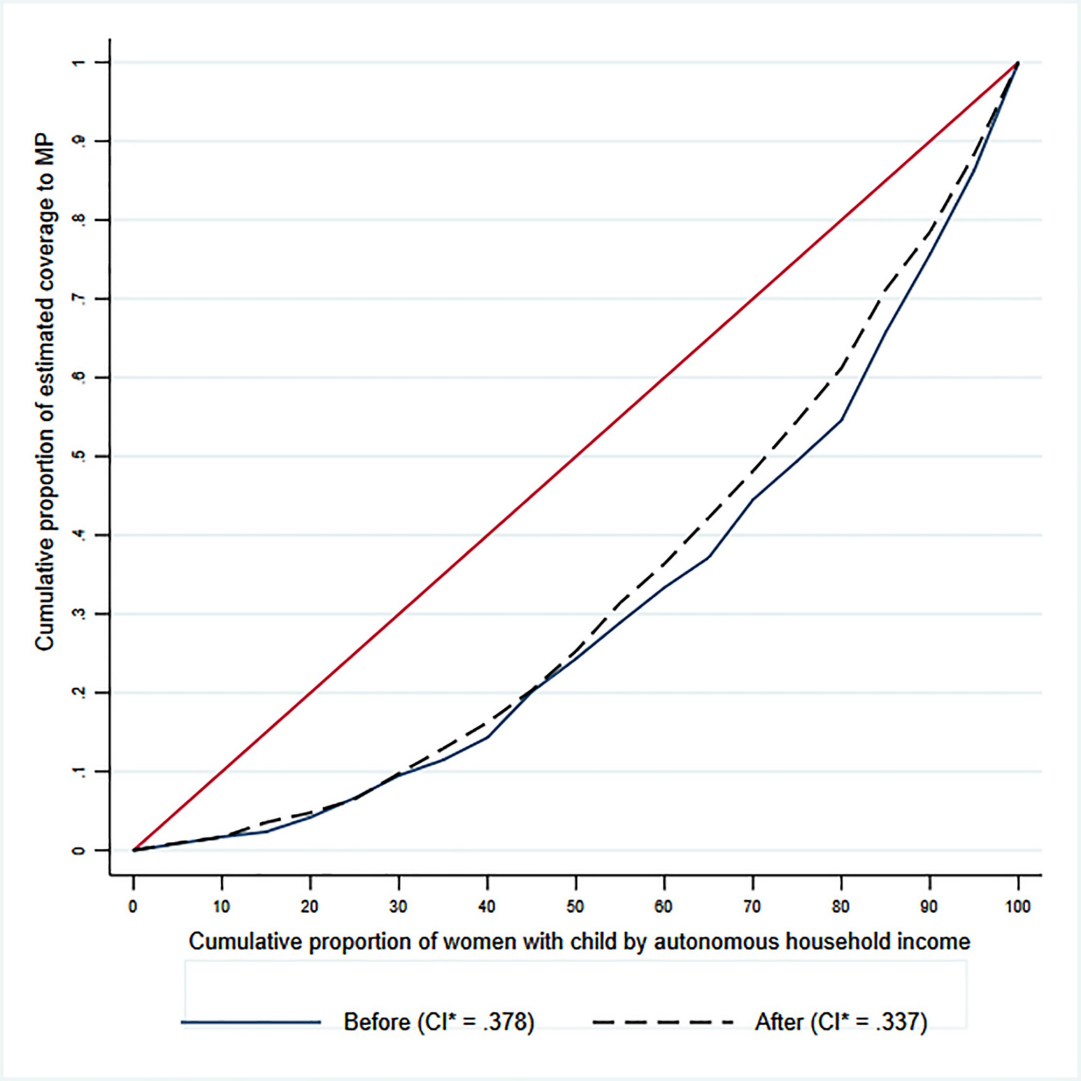
Concentration curve and Concentration Index (CI), years 2009 and 2013.

The Concentration Index before and after the law showed a non-significant reduction in equitable access to MP coverage, as the Concentration Index = 0.38 in 2009 and 0.34 in 2013. This slight reduction favored the middle-income group (between 50% and 90% of the income of the richest population), but the concentration curves overlap (Fig 2). Therefore, after the introduction of the law, the distribution of estimated MP coverage continued to concentrate among the most advantaged women in the country, as measured by ranked household income.

Fig 2 shows the Concentration Curve and the Concentration Index for women who had a live child, by autonomous household income in years 2009 (continuous line) and 2013 (dashed line), i.e., before and after the reform.

### Multivariate analysis

The regression model for year 2009 showed that age, income quintile, and area of residence were associated with a greater likelihood of accessing MP, controlling for the other variables shown in Table 4. The model for year 2013 showed that variables significantly associated with MP access were age, education level, and income quintile. After the reform, highly educated mothers held 5.2 times more access to MP than those with low education.

**Table 4:** Logistic regression models, years 2009 and 2013.

Variables		2009 model, Before Law				2013 model, After Law			
		Odds Ratio	95% CI for Odds Ratio			Odds Ratio	95% CI for Odds Ratio		
			Lower limit	Upper limit	p		Lower limit	Upper limit	p
Age ranges	15–19	1.00				1.00			
	20–29	6.59	2.374	18.299	**	6.30	2.408	16.492	**
	30–39	13.02	4.642	36.543	**	14.75	5.476	39.704	**
	40 and older	22.03	6.587	73.679	**	7.56	2.527	22.627	**
Education level	Low	1.00				1.00			
	Mid	1.67	0.853	3.288		2.86	1.663	4.915	**
	High	1.63	0.804	3.295		5.17	2.843	9.406	**
Income quintile	1 (poorest)	1.00							
	2	3.56	2.019	6.269	**	3.28	2.12	5.084	**
	3	4.88	2.828	8.409	**	6.35	3.823	10.553	**
	4	9.66	5.196	17.973	**	6.71	3.946	11.421	**
	5 (richest)	20.90	9.597	45.52	**	11.04	5.396	22.601	**
Native population	Yes	1.00				1.00			
	No	1.18	0.632	2.19		1.35	0.789	2.323	
Health care system	Public	1.00				1.00			
	Private	1.06	0.527	2.115		1.07	0.636	1.794	
Area of residence	Rural	1.00				1.00			
	Urban	1.80	1.165	2.789	*	1.29	0.832	2.002	
Multidimensional poverty	Non-poor	1.00				1.00			
	Poor	1.22	0.808	1.84		1.43	0.899	2.265	
Constant		4.18	1.339	13.066	**	0.16	0.063	0.421	**
Number of observations		151.186				141.926			

* p<0.05

** p<0.001

In both models, income quintiles were significantly associated with MP use, with steeper gradient between the rich and the poor in 2009. In fact, in 2009, the gradient was 20.9 and it reduced to 11.0 in 2013.

Other variables such as belonging to the native population, having private health care insurance, and multidimensional poverty were not significantly associated with MP access when controlling for the other variables in any of the two models (Table 4).

Finally, in the overall model 2009 and 2013 data combined and controlled for the previously significant variables of age, education level, income quintile, and area of residence, we found that women who had children after the implementation of the Law were 1.28 times more likely to use access MP benefits than those who gave birth in 2009 (Table 5).

**Table 5:** Total logistic regression models, years 2009 and 2013.

Variables		Model before and after the Law			
		Odd Ratio	95% CI for Odds Ratio		
			Lower limit	Upper limit	p
Age ranges	15–19	1.00			
	20–29	6.70	3.182	14.097	**
	30–39	13.72	6.506	28.937	**
	40 and older	13.93	5.943	32.659	**
Education level	Low	1.00			
	Mid	1.88	1.156	3.050	**
	High	2.49	1.512	4.111	**
Income quintile	1 (poorest)	1.00			
	2	3.38	2.381	4.793	**
	3	5.19	3.613	7.455	**
	4	8.57	5.735	12.792	**
	5 (richest)	14.75	9.018	24.113	**
Native population	Yes	1.00			
	No	1.25	0.903	1.724	
Intervention proxy (year)	2009 (before)	1.00			
	2013 (after)	1.28	1.013	1.643	*
Constant		3.88	2.038	7.403	**
Number of women		318.352			

** p<0.001

## Discussion

This study analyzed data for MP coverage in Chile for the period 2000 to 2015. As stated previously, the MP coverage trend was increasing before and after Law 20.545; nevertheless, the slope changed after the Law was implemented. In fact, estimated MP coverage increased from 31.1 in 2009 to 39.5 of mothers in 2013. These numbers account for one of the objectives of the Law: increasing MP coverage in women participating in the labor force. With another source to estimate MP coverage, the CASEN Survey, results were consistent and showed the same general trend.

The Law also aimed to reduce inequities in the distribution of MP benefits among Chilean mothers in the labor force. In this case, results were not as positive. The Concentration Index and Concentration Curve show a non-significant reduction of inequities based on ranked household income. These results suggest that there are no relevant changes in the distribution of maternal protection across different levels of income before and after the implementation of the law. A possible explanation is related to the fact that potential outcomes are not immediate and can only be observed through longer-run effects. Similar findings in this relative range were observed when looking separately at the socioeconomic variables. Additionally, compared by level of education or multidimensional poverty, the gap increased between years 2009 and 2013, at the expense of the most vulnerable working women in the country.

Level of education deserves special analysis. As noted, the access gap between mothers with low and high education levels steepened in the observed years from 2.8 to 5.5 (Table 2). This fact is consistent with multivariate analysis from CASEN data showing that educational level was a significant variable in 2013 but not in 2009; this study revealed that the likelihood of access to MP is 5.17 times higher after the Law for the most educated working mothers compared to those least educated. As context for this observation, we have to take in account that since 2002 in Chile, it is mandatory for every person to have at least 12 years of schooling. As a result, the size of the group of less educated women in 2013 is relatively smaller than in previous years, which may explain some of these findings.

We were unable to locate other research focusing on MP coverage using an equity perspective. Recalling ILO objectives, MP policies should try to harmonize child care and women's paid work, without affecting family health and economic security. This may explain the fact that most studies on MP policies focus on health outcomes of the mother and child, as well as on broader social indicators such as the employment rate.

According to political scenarios for improving population health and reducing inequality [37], Chilean MP reform constitutes a universal social protection policy that improves population outcomes, and most likely, the health of the general population. Nevertheless, according to our study, this Chilean reform did not equally improve equity of access to MP for all mothers, as it showed lower benefit in sub-groups of working mothers, such as women in the poorest income quintile, those who are less educated, and mothers having native background [37, 38]. The Chilean MP reform also left non-working mothers behind.

This reform sought to reduce inequities in key public policies and public health strategies related to care of the mother and child. However, in a country like Chile, characterized by deep socioeconomic stratification, these reforms must be complemented by other policies that can boost their effectiveness, addressing the underlying causes of structural socioeconomic and gender inequalities, such as access to formal occupations and the men-women income gap. In societies with high levels of socioeconomic and gender inequalities, as those in Latin America, the generation, implementation, and evaluation of public policies that aim at reducing inequities must be a political and research priority. When resources must be rationally allocated, it is an ethical, political, and scientific imperative to evaluate the fulfillment of goals intended to improve the life of the population and reduce social and health inequalities.

This study is the first in Chile to assess the impact of the Chilean maternity protection reform on effectiveness and equity indicators. Our results constitute the first evaluation of the objectives and results of Law 20.545. Nevertheless, equity aspects require further research on both working and non-working female populations, as well as on the fathers' participation, responsibility and benefits related to child care.

We identify a number of strengths in this study: (1) it used robust, routinely collected data available in many countries, making feasible the follow up in Chile, as well as comparative studies in and with other countries; (2) it represents a unique empirical evaluation of a globally prioritized public policy in Chile; and (3) it included a number of socioeconomic variables and standard analytical techniques to evaluate the impact of the Chilean MP reform that implemented a particular policy that aimed to achieve equity in access to maternal leave for working mothers.

On the other hand, there are some limitations that we must acknowledge. In a non-controlled before-after design such as this study, changes observed before and after the intervention cannot be attributed to causal relationships [39]. For example, broader factors might also affect our findings, e.g., the fact that women’s participation in the labor force in Chile was moderately rising even before the MP reform. In addition, the CASEN population survey was not designed to estimate MP coverage, which may have introduced some selection and information bias. However, as demonstrated, the data showed that the estimated MP coverage is consistent with observed MP coverage.

## Conclusions

At present, most countries have some form of legislation in place regarding paid maternity or paternity leave, or have established a social security system that provides paid leave to working mothers. The research literature shows that maternity protection policies, especially paid postnatal leaves, can contribute to improving both mothers' and children's health. However, few scientific evaluations of the goals of MP laws and reforms have been conducted. Worldwide, this scarcity of studies is even more acute regarding equity focused evaluations of access to social protection for economically active women who become mothers.

ILO established that worldwide MP policies are mainly focused on protecting women participating in the labor force; so, implicitly, non-working women are not considered, a concern not yet studied. In the case of Chile, these results show that poorly educated young women who are part of labor force, who at the same time belong to the lowest income quintiles, are the most vulnerable group, and national policies are not reaching them.

We found that between 2000 and 2015, mean MP coverage in Chile grew from 24.4% to 44.8%. Using comparable 2009 and 2013 survey data to estimate MP coverage, we observed the same trend, with 31.1% of MP coverage in 2009 escalating to 39.5% in 2013. Generally, there was an increase in MP coverage after the reform, but no significant reduction of inequities in the distribution of MP benefits, particularly among poorer, less educated, and native working women. However, future research could update this analysis by utilizing a longer observation time period following the implementation of the law, in order to explore whether significant differences in estimated MP coverage by socioeconomic status might appear with a longer follow-up period. This study contributes to the scientific literature by providing an empirically-based evaluation of the aims of Chilean Maternity Protection policy reform from both a population-level and an inequality-focused perspective.

## Supporting information

S1 Fig Algorithm to create the variable “Estimated MP coverage”. (TIF)

S1 Table Descriptive statistics of independent variables by MP coverage. (DOCX)

S1 Text RECORD statement. Checklist of items, extended from the STROBE statement that should be reported in observational studies using routinely collected health data.
(DOCX)
