# Supplementary figures and images for: Evaluation of the effectiveness and equity of the maternity protection reform in Chile from 2000 to 2015
Source: PLoS One. 2019 Sep 11;14(9):e0221150. doi: 10.1371/journal.pone.0221150 (PMC6738580; doi:10.1371/journal.pone.0221150)

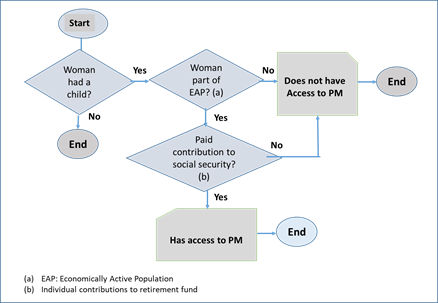

Supplement: S1 Fig — (TIF) [file pone.0221150.s001.tif]
