# Supplementary material for: Evaluation of the effectiveness and equity of the maternity protection reform in Chile from 2000 to 2015
Source: PLoS One. 2019 Sep 11;14(9):e0221150. doi: 10.1371/journal.pone.0221150 (PMC6738580; doi:10.1371/journal.pone.0221150)
Supplement: S1 Table — (DOCX) [file pone.0221150.s002.docx]

Table 1: Descriptive statistics of independent variables by MP Coverage

| **Women** | | **MP Access 2009 (Before Law)** | | | | **MP Access 2013 (After Law)** | | | |
| --- | --- | --- | --- | --- | --- | --- | --- | --- | --- |
|  |  | **Yes** | | **No** | | **Yes** | | **No** | |
|  |  | **N°** | **%** | **N°** | **%** | **N°** | **%** | **N°** | **%** |
| Age range in years | 15-19 | 1,247 | 4.5 | 26,171 | 95.5 | 605 | 4.0 | 14,694 | 96.0 |
|  | 20-29 | 21,73 | 28.7 | 53,966 | 71.3 | 24,133 | 33.6 | 47,795 | 66.4 |
|  | 30-39 | 23,545 | 44.2 | 29,768 | 55.8 | 32,805 | 56.4 | 25,387 | 43.6 |
|  | 40 and older | 3,773 | 47.7 | 4,141 | 52.3 | 3,521 | 39.2 | 5,467 | 60.8 |
| Area of residence | Urban | 47,476 | 32.8 | 97,286 | 67.2 | 56,962 | 41.3 | 80,903 | 58.7 |
|  | Rural | 4,198 | 19.5 | 17,276 | 80.5 | 4,745 | 25.7 | 13,735 | 74.3 |
| Marital status | Yes | 36,507 | 32.8 | 74,712 | 67.2 | 41,592 | 40.4 | 61,42 | 59.6 |
|  | No | 15,167 | 27.6 | 39,85 | 72.4 | 20,115 | 37.7 | 33,218 | 62.3 |
| Ethnicity | Yes | 2,318 | 23.0 | 7,739 | 77.0 | 4,326 | 26.7 | 11,869 | 73.3 |
|  | No | 49,356 | 31.6 | 106,823 | 68.4 | 57,251 | 41.0 | 82,548 | 59.0 |
| Health care system | Public | 35,955 | 26.3 | 100,66 | 73.7 | 42,562 | 33.6 | 84,141 | 66.4 |
|  | Private | 12,73 | 59.9 | 8,527 | 40.1 | 16,993 | 71.0 | 6,941 | 29.0 |
| Education level | Low | 5,351 | 18.8 | 23,183 | 81.2 | 2,017 | 11.2 | 15,964 | 88.8 |
|  | Mid | 24,096 | 25.2 | 71,615 | 74.8 | 24,053 | 30.1 | 55,953 | 69.9 |
|  | High | 22,227 | 52.9 | 19,764 | 47.1 | 35,493 | 61.2 | 22,469 | 38.8 |
| Income quintile | 1 (poorest) | 4060 | 8.6 | 42,995 | 91.4 | 4,586 | 11.1 | 36,826 | 88.9 |
|  | 2 | 10,077 | 24.2 | 31,635 | 75.8 | 12,124 | 31.3 | 26,617 | 68.7 |
|  | 3 | 10,083 | 32.3 | 21,141 | 67.7 | 12,66 | 44.0 | 16,121 | 56.0 |
|  | 4 | 14,309 | 50.8 | 13,863 | 49.2 | 13,606 | 60.1 | 9,042 | 39.9 |
|  | 5 (richest) | 13,145 | 72.7 | 4,928 | 27.3 | 18,731 | 75.6 | 6,032 | 24.4 |
| Multidimensional poverty | Non-poor | 40,381 | 37.6 | 67,095 | 62.4 | 53,695 | 46.7 | 61,285 | 53.3 |
|  | Poor | 10,957 | 20.5 | 42,541 | 79.5 | 6,662 | 19.0 | 28,386 | 81.0 |
| **Total women** |  | **51,674** | **31.1** | **114,562** | **68.9** | **61,707** | **39.5** | **94,638** | **60.5** |
