# Supplementary material for: Evaluation of the effectiveness and equity of the maternity protection reform in Chile from 2000 to 2015
Source: PLoS One. 2019 Sep 11;14(9):e0221150. doi: 10.1371/journal.pone.0221150 (PMC6738580; doi:10.1371/journal.pone.0221150)
Supplement: S1 Text — Checklist of items, extended from the STROBE statement that should be reported in observational studies using routinely collected health data. (DOCX) [file pone.0221150.s003.docx]

**The RECORD statement – checklist of items, extended from the STROBE statement that should be reported in observational studies using routinely collected health data.**

|  | **Item No.** | **STROBE items** | | **Location in manuscript where items are reported** | **RECORD items** | **Location in manuscript where items are reported** | **Details or extracts from manuscript** |
| --- | --- | --- | --- | --- | --- | --- | --- |
| **Title and abstract** | | | | | | | |
|  | 1 | (a) Indicate the study’s design with a commonly used term in the title or the abstract (b) Provide in the abstract an informative and balanced summary of what was done and what was found | | Abstract and Methods | RECORD 1.1: The type of data used should be specified in the title or abstract. When possible, the name of the databases used should be included.  RECORD 1.2: If applicable, the geographic region and timeframe within which the study took place should be reported in the title or abstract.  RECORD 1.3: If linkage between databases was conducted for the study, this should be clearly stated in the title or abstract. | RECORD 1.1: The type of data and name of the databases uses are provided in the Abstract, as well as the Method.  RECORD 1.2  Time study is provided in Abstract and Method  RECORD 1.3  We linked dataset across years before and after the reform | In this study, we performed a non-experimental, before-after intervention design assessing the impact of the maternity protection (MP) reform on effectiveness and equity indicators. The intervention was the implementation of Chilean Law 20.545 in 2011, which modified the requisites and benefits associated with maternity protection policies in Chile.  We used individual level data from three different sources:   1. Social Security Superintendence (SUSESO): Postnatal maternal leaves during between 2000 and 2015 (59,229 in 2000 to 108,462 in 2015). See Figure 1. 2. Department of Health Care Statistics and Information: Women who gave a live birth between 2000 and 2015 (approx. 240,000 live birth each year). See figure 1. 3. Socioeconomic Characterization Survey 2009 and 2013 waves (CASEN): Estimated MP coverage and inequities in MP benefits (166,236 and 157,345, respectively). See Table 2   **Our Findings:**   1. The MP coverage increased from 24.4% to 44.8%. The mean annual increase before implementing the law was 3.5%, while after 2011 it rose to 5.8%. 2. The “Estimated MP coverage” improved from 31.1% to 39.5% (p<0.001) between 2009 and 2013. |
| **Introduction** | | | | | | | |
| Background rationale | 2 | Explain the scientific background and rationale for the investigation being reported | | Introduction |  | Introduction  Paragraphs 1-3 | In the scientific literature, there is agreement regarding the impact of maternal leave on mothers' health; for instance, longer prenatal and postnatal leaves are associated with better physical and mental health in mothers, the length of breastfeeding, and children’s health and development. Also, there is some evidence about the possible impact of MP reforms on broader social indicators such as employment, income, and women's fertility rates.  To our knowledge, there are no other papers focusing in MP coverage using an equity perspective. |
| Objectives | 3 | State specific objectives, including any prespecified hypotheses | | Last paragraph of introduction |  |  | This study aims to assess the two main objectives of the MP law of 2011 in Chile   1. to increase maternity protection coverage (effectiveness) 2. to reduce inequities in access to the benefits (equity) |
| **Methods** | | | | | | | |
| Study Design | 4 | Present key elements of study design early in the paper | | Methods |  |  | Using administrative data, we constructed an indicator of the MP coverage between 2000 and 2015. Additionally, we used a national survey before and after the law to estimate MP coverage.  For the analysis of inequalities, we used a set of techniques:   1. bivariate analysis between “Estimated MP Coverage” and each of the independent variables using Chi-squared or Student's t-test for independent samples; 2. absolute inequality gaps between the years 2009 and 2013 and relative gaps between the extreme values of the socioeconomic variables; and 3. estimation of the Concentration curve and Concentration index (CI), two indicators that assessed global inequity access to MP. |
| Setting | 5 | Describe the setting, locations, and relevant dates, including periods of recruitment, exposure, follow-up, and data collection | | Methods |  |  | The population studied comprised women who gave birth to a live newborn between the years 2000 and 2015 in Chile. The administrative data (DEIS) comprises information of all new born in Chile between 2000 and 2015. Our second data source (SUSESO) includes information about every woman with maternal leave after the birth in each year in Chile.  We used the household survey CASEN 2009 and 2013. The survey provides information on demographics, socioeconomic, fertility, and health characteristics from a national representative sample of households using a probabilistic, multistage, and stratified design. 246,924 and 218,491 individuals were surveyed in 2009 and 2013, respectively.  Variables of interest were homologated in both years, and final datasets were appended to implement the proposed methodology. |
| Participants | 6 | *(a) Cohort study* - Give the eligibility criteria, and the sources and methods of selection of participants. Describe methods of follow-up  *Case-control study* - Give the eligibility criteria, and the sources and methods of case ascertainment and control selection. Give the rationale for the choice of cases and controls  *Cross-sectional study* - Give the eligibility criteria, and the sources and methods of selection of participants  *(b) Cohort study* - For matched studies, give matching criteria and number of exposed and unexposed  *Case-control study* - For matched studies, give matching criteria and the number of controls per case | | Methods | RECORD 6.1: The methods of study population selection (such as codes or algorithms used to identify subjects) should be listed in detail. If this is not possible, an explanation should be provided.  RECORD 6.2: Any validation studies of the codes or algorithms used to select the population should be referenced. If validation was conducted for this study and not published elsewhere, detailed methods and results should be provided.  RECORD 6.3: If the study involved linkage of databases, consider use of a flow diagram or other graphical display to demonstrate the data linkage process, including the number of individuals with linked data at each stage. | RECORD 6.1: Details of the sample selection are provided in Methods section. See details in next column  RECORD 6.1: Selection criteria and results are provided in the next column.  RECORD 6.3 We compared data from different sources on each year for robustness purposes, additionally we appended data across years in objective 1 and 2. | To answer the first research goal (effectiveness), we used information of all newborns and every woman with maternal leave after the birth between 2000 and 2015 in Chile.  To answer the second goal (equity), we explored the number of women aged 15 to 49 with hospital discharge for vaginal delivery or caesarean section. This number was 166,236 and 157,345 in 2009 and 2013, respectively.  Individuals were classified in two groups based on:   1. With access to MP coverage: women who are part of the labour force and contributed to social security the month prior to participation in the survey. They included 51,674 and 61,707 in 2009 and 2013, respectively; 2. Without access to MP coverage: women who are not in the labour force or did not contribute to social security during the month prior to participation in the survey. They included 114,562 and 94,638 in 2009 and 2013, respectively   See Table 1 |
| Variables | 7 | Clearly define all outcomes, exposures, predictors, potential confounders, and effect modifiers. Give diagnostic criteria, if applicable. | | Methods  Paragraph “Study dependent variable” and “Study independent variables” | RECORD 7.1: A complete list of codes and algorithms used to classify exposures, outcomes, confounders, and effect modifiers should be provided. If these cannot be reported, an explanation should be provided. | See Methods: section Independent and dependant variables. For a summary, see next column | The main outcome was MP coverage, defined as the number of women who had a paid postpartum leave in a certain year, divided by the number of women who gave birth to a live child the same year. There are two estimations for MP coverage, which is the dependent variable.  The “MP coverage” was calculated with official databases from SUSESO and DEIS. The “Estimated MP coverage” was created using CASEN survey data, following the procedure below:   - 1. Value “1” was assigned to the women fulfilling three condition: hospital discharge for vaginal delivery or caesarean section, and are part of the labour force and contributed to social security during the month prior to participation in CASEN survey.   2. The value “0” was assigned to women who, having had a hospital discharge for delivery. Are not part of the labor force and/or did not contribute to social security during the month prior to participation in the CASEN survey.   **Study independent variables (Predictors)**  *Sociodemographic:*   - Age groups: “15-19 years”, “20-29 years”, “30-39 years”, “40 years and older”; - Area of residence: “urban”, “rural”; - Marital status: “Yes” (married / cohabiting), “No” (single / separated / divorced / widowed); - Ethnicity: “Yes”, “No”.   *Socioeconomic:*   - Type of Health insurance: “Public”, “Private”; - Education level: “Primary” (<=8 years), “Secondary” (9-12), “Higher” (>=13 years). - Household income quintile: “1 (poorest)”, “2”, “3”, “4”, and “5 (richest)”. - Multidimensional poverty: “Yes”, “No”. Composite indicator with four dimensions (Education, Work and Social Security, Health, and Housing domains) and several indicators within each dimension. |
| Data sources/ measurement | 8 | For each variable of interest, give sources of data and details of methods of assessment (measurement).  Describe comparability of assessment methods if there is more than one group | | Methods |  |  | To assess MP coverage, we used the number of postnatal maternal leaves during years 2000-2015 that were officially published by the Social Security Superintendency (Superintendencia de Seguridad Social, SUSESO)  To estimate the number of women who gave a live birth, multiple births were adjusted to one woman using official data from the Department of Health Care Statistics and Information (Departamento de Estadísticas e Información en Salud, DEIS), at the Ministry of Health  To analyze inequities in MP benefits, we used information from the National Socioeconomic Characterization Survey (CASEN), which provides information on the socioeconomic level of the Chilean population. |
| Bias | 9 | Describe any efforts to address potential sources of bias | | Discussion |  |  | To deal with potential bias of the before and after design, we included in the multivariate model a proxy of intervention. This implies that controlling for other socioeconomic variables, we are able to assess the impact of the reform in a group of women with similar characteristics.  The CASEN population survey was not designed to estimate MP coverage; however, the estimated MP coverage is consistent with administrative information. |
| Study size | 10 | Explain how the study size was arrived at | | Methods |  |  | Objective 1: The population studied is comprised of women who gave birth to a live newborn between the years 2000 and 2015. In Chile, over 99% of births occur in a medical facility in Chile and registration is compulsory. We obtained those records from administrative sources, namely the Social Security Superintendency (SUSESO) and Department of Health Care Statistics and Information (DEIS), at the Ministry of Health.  Objective 2: The CASEN survey employs multistage probabilistic sampling, stratified by urban and rural area. Samples were independent from each other. The inclusion criteria for selection of counties in both years were: (i) all urban counties with over 40 000 inhabitants, (ii) all rural counties irrespective of the number of inhabitants, (iii) a random selection of a small proportion of counties with less than 40 000 inhabitants. Fourteen and seventeen hard to reach counties were excluded from the 2000 and 2013 surveys respectively because of their very difficult geographical access. Within each  county, households were randomly selected.  The survey includes information for all women; we selected those women who were pregnant during the last year. The final sample was 2.434 and 2.088 individuals each year who represent 166,236 and 157.345 weighted cases at the national level in 2009 and 2013, respectively. |
| Quantitative variables | 11 | Explain how quantitative variables were handled in the analyses. If applicable, describe which groupings were chosen, and why | | Methods |  |  | See item 7 |
| Statistical methods | 12 | (a) Describe all statistical methods, including those used to control for confounding  (b) Describe any methods used to examine subgroups and interactions  (c) Explain how missing data were addressed  (d) *Cohort study* - If applicable, explain how loss to follow-up was addressed  *Case-control study* - If applicable, explain how matching of cases and controls was addressed  *Cross-sectional study* - If applicable, describe analytical methods taking account of sampling strategy  (e) Describe any sensitivity analyses | | Methods: Data Analysis |  |  | The “MP Coverage” was calculated for every year from 2000 to 2015. The “Estimated MP coverage” was calculated for the years 2009 and 2013, respectively, after appending the data. Inequities were analyzed through several procedures: 1) bivariate analysis between “Estimated MP Coverage” and each of the independent variables, analyzing data before and after the law enforcement using Chi-squared or Student's t-test for independent samples; 2) absolute inequality gaps between the years 2009 and 2013 and relative gaps between the extreme values of the socioeconomic variables; and 3) estimation of the Concentration Curve and Concentration Index (CI), two indicators that assessed global inequity access to MP. This exploration of data is more robust than the single gap analysis, since it evaluates inequities through the socioeconomic gradient. 4) multivariate logistic models, adjusting two models, one for the year 2009 and another for 2013. In both models, the dependent variable was “Estimated MP Coverage”, while sociodemographic and socioeconomic variables were the independent variables. 5) Finally, evaluation of the likelihood of accessing MP before and after the reform, by estimating a final multivariate logistic model for years 2009 and 2013 combined, including all significant variables that were found in the previous models. In this final model, a unique covariable, acting as “Intervention proxy”, was included, assigning value “0” for women who had given birth in 2009 (pre-reform) and “1” for those who gave birth in 2013 (post-reform).  All analyses were conducted applying standardized weights, based on the complex sampling procedure of CASEN survey |
| Data access and cleaning methods |  |  | | Ending Section | RECORD 12.1: Authors should describe the extent to which the investigators had access to the database population used to create the study population.  RECORD 12.2: Authors should provide information on the data cleaning methods used in the study. | We have full access to anonymized individual records from public records and survey.  See next column | Databases used in this study are anonymous and their use does not require informed consent.  Datasets can be obtained directly from the Social Development Ministry and the Ministry of Health. (<http://observatorio.ministeriodesarrollosocial.gob.cl/casen/casen_obj.php>)  (<http://www.deis.cl/bases-de-datos-nacimientos/>).  Objective 1: We collapsed individual level data of live births for each year to adjust multiple births as per one per one woman.  Then, we collapsed yearly information in one dataset for all periods. We validated our data by contrasting our results with the official public information available.  Objective 2: We homogenized variables across surveys. We use internal consistency methods, such us pregnancy, gender, and fertile age (15-49) in each survey. Then different years were appended. |
| Linkage |  | .. | |  | RECORD 12.3: State whether the study included person-level, institutional-level, or other data linkage across two or more databases. The methods of linkage and methods of linkage quality evaluation should be provided. |  | See previous section. |
| **Results** | | | | | | | |
| Participants | 13 | (a) Report the numbers of individuals at each stage of the study (*e.g.*, numbers potentially eligible, examined for eligibility, confirmed eligible, included in the study, completing follow-up, and analysed)  (b) Give reasons for non-participation at each stage.  (c) Consider use of a flow diagram | | Methods | RECORD 13.1: Describe in detail the selection of the persons included in the study (*i.e.,* study population selection) including filtering based on data quality, data availability and linkage. The selection of included persons can be described in the text and/or by means of the study flow diagram. | See point 6 and 10 | See point 6 and 10 |
| Descriptive data | 14 | (a) Give characteristics of study participants (*e.g.*, demographic, clinical, social) and information on exposures and potential confounders  (b) Indicate the number of participants with missing data for each variable of interest  (c) *Cohort study* - summarise follow-up time (*e.g.*, average and total amount) | | Results and Supporting information 1 |  |  | See table S1 table: Maternal protection access (weighted number and percentage) by socioeconomic characteristics for descriptive statistics  See also Table 2: Estimated Maternity Protection Coverage according to sociodemographic variables (2009 and 2013) and Table 3: Estimated Maternity Protection Coverage according to socioeconomic variables (2009 and 2013). |
| Outcome data | 15 | *Cohort study* - Report numbers of outcome events or summary measures over time  *Case-control study* - Report numbers in each exposure category, or summary measures of exposure  *Cross-sectional study* - Report numbers of outcome events or summary measures | | Results |  |  | See Table 2: Official and Estimated Maternity Protection (MP) Coverage and Average Age according to MP coverage  Figure 2: The Lorenz Curve and Concentration Index (CI), years 2009 and 2013, show the concentration curve and the concentration index for women who had a live child, by income quintiles in years 2009 (continuous line) and 2013 (dashed line) before and after the reform. |
| Main results | 16 | (a) Give unadjusted estimates and, if applicable, confounder-adjusted estimates and their precision (e.g., 95% confidence interval). Make clear which confounders were adjusted for and why they were included  (b) Report category boundaries when continuous variables were categorized  (c) If relevant, consider translating estimates of relative risk into absolute risk for a meaningful time period | | Results |  |  | Objective 1: Not required. We worked with the entire group of pregnant Chilean women in each year.  Objective 2: All estimates have 95% CI. Given the structure of the survey, to provide unweighted results would be incorrect.  Table 4: Logistic regression models, years 2009 and 2013 provide risk ratios before and after the law by age ranges, education level, Income quintile, ethnic group affiliation, health care system, area of residence, and multidimensional poverty  Age and schooling were categorized as described in section 7 above. Income was categorized in quintiles for interpretative purposes. |
| Other analyses | 17 | Report other analyses done—e.g., analyses of subgroups and interactions, and sensitivity analyses | |  |  |  | We used internal and external validation of our variables. For instance, in spite of using different data sources, it should be noted that MP Coverage is within the range of the CI for Estimated MP Coverage, representing a fairly similar estimate compared to the MP coverage gold standard. Additionally, we performed a sensibility analysis to check the robustness of our results to changes in the operationalization of variables and their categorization. |
|  | | | **Discussion** | | | | |
| Key results | 18 | Summarise key results with reference to study objectives | | Discussion |  |  | The study analyzed data for MP coverage for the period 2000 to 2015. We found an increasing trend in MP coverage taking place in the country before the law was implemented, but that accelerated after the MP reform. In fact, the mean annual growth of MP coverage was 3.5% annually, and it grew to 5.8% after 2011. These numbers account for one of the objectives of the law: incrementing MP coverage in women participating in the EAP. Using another source to estimate MP coverage, with the CASEN Survey, data was consistent and showed the same general trend.  The law also aimed to reduce inequities in the distribution of MP benefits among Chilean mothers in EAP. In this case, results were not so positive. The concentration index and curve show a non-significant reduction of inequities based on household income. Similar findings in relative inequity gaps were observed when looking at socioeconomic variables separately. For instance, in the case of level of education or multidimensional poverty, the gap increased between years 2009 and 2013, at the expense of the most vulnerable working women in the country. |
| Limitations | 19 | Discuss limitations of the study, taking into account sources of potential bias or imprecision. Discuss both direction and magnitude of any potential bias | | Discussion | RECORD 19.1: Discuss the implications of using data that were not created or collected to answer the specific research question(s). Include discussion of misclassification bias, unmeasured confounding, missing data, and changing eligibility over time, as they pertain to the study being reported. | CASEN survey was not designed to estimate MP coverage, however, given the size of the interviewed population and the consistency with routinely information, we could reject sample selection bias. See next column | There were limitations to our study. First, as in any non-experimental study, there is the potential for unmeasured cofounding. However, we controlled for potential cofounding by individual and household characteristics.  In a non-controlled before-after design such this study, changes observed before and after the intervention cannot be attributed to causal relationships.  In addition, the CASEN population survey was not designed to estimate MP coverage, which may have introduced some selection and information bias. However, as noted, the data showed that the estimated MP coverage is consistent with MP coverage. |
| Interpretation | 20 | Give a cautious overall interpretation of results considering objectives, limitations, multiplicity of analyses, results from similar studies, and other relevant evidence | | Discussion |  |  | Most studies of MP policies focus on health outcomes of the mother and child, as well as on broader social indicators such as the employment rate.  Our study focused on a new reform in a developing country with the lowest female employment rate in Latin America, informal labour markets, and a feeble welfare system. Our results were obtained using the whole universe of pregnant women (objective 1) and one of the largest, and long standing, household surveys in the developing world. |
| Generalisability | 21 | Discuss the generalisability (external validity) of the study results | | Discussion |  |  | Other countries, with similar surveys, could estimate coverage at the national level and also assess inequity.  Additionally, the parallel use of routinely collected (administrative) and survey data could provide a more precise understanding of the phenomena as a whole.  This study provides the basis for and enables the generation, discussion, approval, implementation, and evaluation of public policies that aim at reducing inequities, and this must be a political and research priority. |
| **Other Information** | | | | | | | |
| Funding | 22 | Give the source of funding and the role of the funders for the present study and, if applicable, for the original study on which the present article is based | | Ending Section |  |  | This study was funded by the Research Fund of Universidad del Desarrollo, year 2015 <http://fondosconcursables.udd.cl/fondos-internos-udd/>. The funders had no role in study design, data collection and analysis, decision to publish, or preparation of the manuscript. |
| Accessibility of protocol, raw data, and programming code |  | .. | | Ending Section | RECORD 22.1: Authors should provide information on how to access any supplemental information such as the study protocol, raw data, or programming code. | Once accepted, the programming code will be available online. Other sources (questionnaires, methods and datasets) are available on the column on the right. | Databases used in this study are anonymous and their use does not require informed consent. Datasets can be obtained without any restriction from  Social Development Ministry http://observatorio.ministeriodesarrollosocial.gob.cl/casen/casen_obj.php  Ministry of Health websites <http://www.deis.cl/bases-de-datos-nacimientos/>  Social Security Superintendency  http://www.suseso.cl/607/w3-propertyvalue-10362.html |
